# The Role of Chitosan and Gelatin-Based Scaffolds in Bone Regeneration: A Systematic Review

**DOI:** 10.7759/cureus.69793

**Published:** 2024-09-20

**Authors:** Vyshnavi B Sindhusha, Jayakumar N Doraiswamy

**Affiliations:** 1 Periodontics, Saveetha Dental College and Hospitals, Saveetha Institute of Medical and Technical Sciences, Saveetha University, Chennai, IND

**Keywords:** bone regeneration, chitosan, gelatin, polymers, scaffold

## Abstract

Guided bone regeneration facilitates the growth of new bone in areas where there is a bone defect or insufficiency. This technique involves placing a barrier membrane over the bone graft site, and the membrane prevents the invasion of soft tissue (such as gingival tissue) into the bone graft area. This allows the slower-growing bone cells to populate the area without competition, promoting proper bone regeneration. When combined, chitosan and gelatin create composite scaffolds that leverage the strengths of both materials. Chitosan provides structural integrity and antimicrobial properties, and gelatin enhances cell attachment and proliferation, which improves mechanical properties and makes it more suitable for supporting bone regeneration in load-bearing areas. This systematic review aims to evaluate the effectiveness of chitosan and gelatin-based scaffolds in bone regeneration.

Various databases such as PubMed, Cochrane Library, LILAC, and Google Scholar were screened to adhere to the eligibility criteria. The included studies in the review were the in vivo and in vitro assessment of the chitosan and gelatin efficiency as a scaffold. Six studies were investigated for the involvement of chitosan and gelatin-based scaffolds in bone regeneration. Of these, two in vivo studies examined bone regeneration by measuring alkaline phosphatase activity (ALP) using different staining techniques, while the remaining four in vitro studies used histologic and histometric analysis where stem cells, chemicals, and other biopolymers were compared.

Chitosan and gelatin scaffolds consistently showed better results in terms of bone repair throughout all six experiments. Gelatin's capacity for regeneration can be increased by mixing it with chitosan. For additional advancement, future researchers need to focus on incorporating biopolymers. The potential of scaffolds composed of gelatin and chitosan to replace tissue lost due to periodontitis shows great clinical significance.

## Introduction and background

Guided bone regeneration (GBR) in dentistry is being used in several specialities of dentistry in addition to periodontics. These include oral surgery, implantology and also for segmental bone defect regeneration [1]. In periodontics, regeneration is the process of rebuilding the bone, cementum and periodontal ligament structures that support the teeth that may have been destroyed or impaired by periodontal disease or trauma [2]. In order to encourage and facilitate the regeneration of periodontal tissues, the technique usually consists of a combination of progenitor cells (such as stem cells) together with biocompatible materials or scaffolds, frequently supplemented with bioactive chemicals [3].

Three-dimensional structures known as scaffolds are made of biocompatible materials that resemble the extracellular matrix (ECM) seen in natural tissue. Before eventually integrating with the surrounding tissue, they serve as transient templates to direct the growth and organization of the tissue. The choice of scaffold biomaterial depends on the type of tissue, degradation rate and interaction with the cells and surrounding tissue [4]. Scaffolds are mainly divided into natural-based polymers and synthetic-based polymers. These two groups are appropriate for diverse applications in bone tissue engineering because of their distinct qualities and attributes [5]. Polylactic acid and poly lactic-co-glycolide are examples of synthetic-based polymers. Chitosan, alginate, gelatin and silk strands are examples of natural-based polymers. Because they offer an environment that is conducive to cell adhesion, proliferation and differentiation all essential for effective tissue regeneration, natural-based polymers are advantageous [6].

Enhancing bone regeneration by the combination of biomaterials and other bioactive compounds has demonstrated significant promise. Mesenchymal stem cells (MSCs) are multipotent stem cells that can be derived from bone marrow, adipose tissue and the umbilical cord. Their many characteristics include immunosuppression and multipotent differentiation [7]. Naturally occurring proteins called bone morphogenetic proteins (BMPs) stimulate MSCs to differentiate into osteoblasts, which promote the production of new bone. In regions with bone abnormalities or injuries, BMPs can greatly accelerate bone growth and improve the regeneration process when combined with scaffolds and MSCs [8].

Chitosan is a naturally occurring polysaccharide that is produced when chitin is deacetylated. It structurally resembles glycosaminoglycans (the exoskeleton). Because of its ability to support tissue regeneration and repair, it has drawn a lot of attention in the field of regenerative medicine [9]. Chitosan is available in various forms, such as sponges, hydrogels, films and fibres. It can serve as a two-dimensional or three-dimensional scaffold for wound dressing and tissue engineering processes. It is known for its biodegradability, nontoxic nature and hydrophilic surface, which facilitates cell adhesion and proliferation. Its mucoadhesive properties allow it to adhere to mucosal surfaces for localised therapeutic effects [10].

Gelatin is a cross-linked collagen, and the cross-linking helps in structural stabilisation. It is extensively employed in several biomedical domains, including tissue engineering. Because gelatin scaffolds can replicate the ECM of natural tissues and are biocompatible and biodegradable, they are frequently utilised in tissue engineering [11]. Over time, proteases have the ability to enzymatically break down gelatin, resulting in the formation of new tissue. The cross-linked gelatin scaffold preserves functionality and helps in the controlled release of growth factors (GFs) and other bioactive molecules at the site of implantation, thus stimulating cellular activities to promote tissue regeneration [12]. This systematic review aims to assess the efficacy of scaffolds based on gelatin and chitosan in bone healing.

## Review

Aim and objective

The aim of this systematic review is to evaluate the effectiveness of chitosan and gelatin-based scaffolds in bone regeneration for the treatment of periodontal intrabony defects. The objective of this systematic review is to review the literature to obtain an updated overview of the effectiveness and use of chitosan and gelatin-based scaffolds in bone regeneration.

Materials and methods

Protocol and Registration

**Figure 1:**
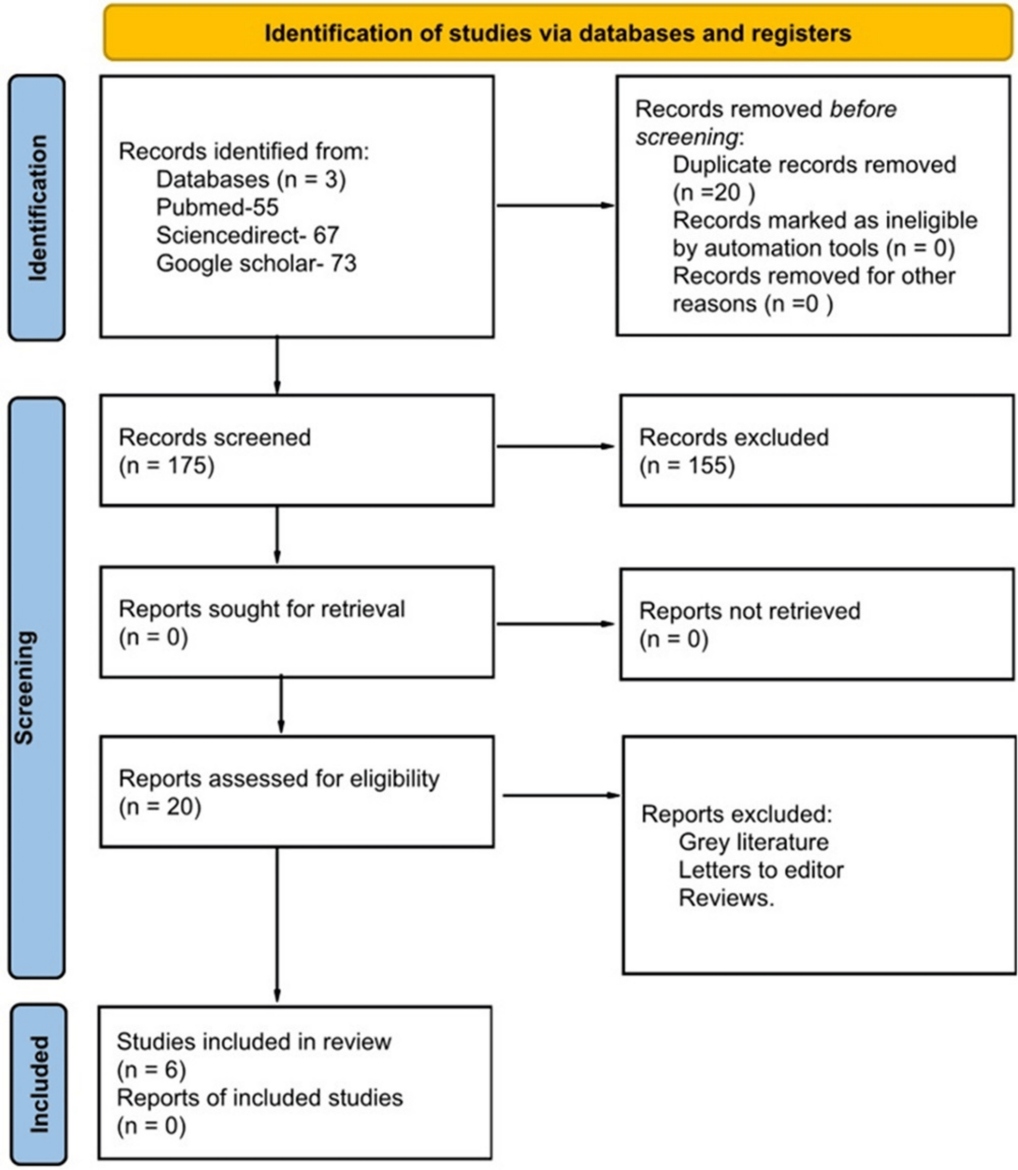
PRISMA chart PRISMA: Preferred Reporting Items for Systematic Reviews and Meta-Analysis

The Preferred Reporting Items for Systematic Reviews and Meta-Analysis (PRISMA) guidelines offered a precise process that was followed in selecting the systematic review's methods and inclusion criteria (Figure 1). This study was registered in the International Prospective Register of Systematic Reviews (PROSPERO; registration number: CRD42023450552).

Focus Question

Does chitosan and gelatin-based scaffold have the osteogenic potential to promote bone regeneration?

Population, Intervention, Comparison and Outcome (PICO) Analysis

The PICO analysis was used as follows: population (P) - osseous defects or intrabony defects, intervention (I) - chitosan and gelatin-based scaffolds, comparison (C) - vacant or unfilled bone defects where no intervention is needed, and outcome (O) - bone regeneration.

Search Strategy

A comprehensive literature review was performed employing the PubMed, Cochrane Library, LILACS and Google Scholar databases. The most recent research (up to March 2023) regarding the application and efficacy of chitosan and gelatin-based scaffolds for bone regeneration was chosen. There were no limitations on the kinds of biomaterials, chemicals or biological parts that were mixed with gelatin and chitosan.

Medical Subject Headings (MeSH) Terms

(((Chitosan [Text Word] AND (gelatin) [Text word])) AND (scaffold [Text Word])) AND (bone regeneration [Text Word])) OR (oral bone regeneration [Text Word]) AND (‘ periodontal engineering’).

Screening and Selection

A thorough search of the literature was done utilising databases and targeted keywords to find pertinent papers about bone regeneration. Every article that was discovered during the literature search had its titles and abstracts examined by two impartial reviewers (V.S. and N.D.J.) with a kappa score (0.97). After that, they examined the complete texts of any papers that may qualify to see if they fulfilled the predetermined standards for inclusion and exclusion. These researchers then extracted the relevant information from each eligible article. The extracted information includes details about the study design (e.g. randomised controlled trial), whether the study involved in vivo or in vitro analysis and the specific types of scaffold biomaterials used for bone regeneration.

Risk of Bias Assessment

The risk of bias for the studies was calculated using the Quinn tool (for in vitro studies) and SYRCLE (for in vivo studies) which are mentioned in Tables 1-2. The Quinn tool was evaluated using the following parameters such as sample size, methodology, randomisation, blinding and presentation of results, whereas the SYRCLE tool was evaluated based on single operator protocol and sample standardisation.

**Table 1:** Risk of assessment using the Quinn tool (in vitro studies) >70% - low risk of bias; 50-70% - medium risk of bias; <50% - high risk of bias

Criteria	Ge et al. (2012) [13]	Song et al. (2014) [14]	Oryan et al. (2016) [15]	Zang et al. (2016) [16]	Zhou et al. (2017) [17]	Sukpaita et al. (2019) [18]
Clearly stated aims/objectives?	2	2	2	2	2	2
Detailed explanation of sample size calculation?	2	2	2	2	2	2
Detailed explanation of the sampling technique?	2	1	2	2	2	2
Details of comparison group?	2	2	2	2	1	2
Detailed explanation of methodology?	2	2	2	2	2	2
Operator details?	2	2	2	2	1	2
Randomization?	2	2	1	2	1	1
Method of measurement of outcome?	2	1	2	2	2	2
Outcome assessor details?	1	2	2	2	1	2
Blinding?	2	2	2	1	2	1
Statistical analysis?	2	2	2	2	2	2
Presentation of results?	2	2	2	2	2	2

**Table 2:** SYRCLE tool for risk of bias (in vivo studies) SYRCLE: SYstematic Review Centre for Laboratory animal Experimentation

Criteria	Ge et al. (2012) [13]	Song et al. (2014) [14]	Oryan et al. (2016) [15]	Zang et al. (2016) [16]	Zhou et al. (2017) [17]	Sukpaita et al. (2019) [18]
Random group allocation (selection)	Yes	Yes	Unclear	Yes	Yes	Yes
Groups similar at baseline (selection)	Yes	Yes	Yes	Yes	Yes	Yes
Blinded group allocation (selection)	Yes	Yes	Yes	Yes	Yes	Yes
Random housing (performance)	Yes	No	Yes	No	Yes	Unclear
Blinded interventions (performance)	Unclear	Yes	Yes	No	Yes	Yes
Random outcome assessment (detection)	Yes	Yes	Yes	Yes	Yes	Yes
Blinded outcome assessment (detection)	Yes	Yes	Unclear	No	Yes	Yes
Selective outcome reporting bias	Yes	No	Yes	Yes	Yes	Yes
Attrition bias	No	No	Yes	No	No	No
Other biases	No	No	Unclear	No	Unclear	No

Data Extraction

The data from the included studies was extracted, assessed and entered into the electronic spreadsheet which includes author and year, type of study, groups, and statistics along with the results of individual studies (Table 3).

**Table 3:** Characteristics of included studies HGCCS: nano hydroxyapatite-coated genipin-chitosan conjugation scaffold; PDLSCs: periodontal ligament stem cells; CFB-HAP: chitosan/fibroin-hydroxyapatite; C/ABB: chitosan/anorganic bovine bone; hJBMMSCs: human jaw bone marrow-derived mesenchymal stem cells; WH: Whitelock; HAP: hydroxyapatite; CS/DA: chitosan/dicarboxylic acid; hPDLCs: human periodontal ligament cells; H&E: hematoxylin and eosin; ELISA: enzyme-linked immunosorbent assay; BV: bone volume; BMD: bone mineral density; ALP: alkaline phosphatase activity; micro-CT: micro-computed tomography; ANOVA: analysis of variance

Author and year	Type of study	Groups	Methodology	Parameters and statistics	Results
Ge et al. (2012) [13]	In vitro and in vivo study (rat calvarial defects)	Group 1: PDL stem cell-seeded chitosan scaffold; Group 2: vacant defects	Eighteen adult male Sprague-dawley rats each eight-week-old and weighing 180-220 grams were randomly assigned to one of three groups (n=6) and given one of three treatments: GCF + PDLSC, HGCCS + PDLSC, no cells and no scaffold (negative control group). The animals were put under anaesthesia and killed after 12 weeks.	Immunohistochemistry: STRO-1 (mesenchymal stem cell marker) increased in group 1 H&E staining: new bone formation; one-way analysis of variance (ANOVA)	PDLSCs tested positive for STRO-1 and were colonogenic. They were able to differentiate into adipocytes and osteoblasts in vitro. Utilizing PDLSC-seeded HGCCS encouraged calvarial repairing of the bones.
Song et al. (2014) [14]	In vitro and in vivo study (54 rats- calvarial defects)	Group 1: chitosan + hydroxyapatite Group 2: normal wound healing	There were 54 rats in the study and by employing a trephine bur, a circular bony defect with an 8 mm diameter was created in the calvaria center. Thermally infused separation was used to prepare the CFB–HAP membrane. The bone defect was covered by CFB–HAP membrane in the experimental group (n518) and by a resorbable collagen membrane (Bio-Gide) in the control group (n518). No membrane was used in the negative control group (n518). Six animals were euthanized in each group at two, four and eight weeks following surgery.	Bone volume/total volume: micro-CT analysis is more in group 1; H&E staining; new bone formation is increased in group 1; post hoc t-test	The membrane groups and the negative control had significantly different bone volume (BV) and bone mineral density (BMD) (P<0.05). Nonetheless, no discernible variations were found between the collagen group and the CFB-HAP group. Our conclusion was that there is a great deal of promise for CFB-HAP as a guided bone regeneration (GBR) membrane.
Oryan et al. (2016) [15]	In vitro and in vivo study (25 wistar rat-calvarial defects)	Group 1: chitosan; Group 2: gelatin; Group 3: chitosan + gelatin; Group 4: normal wound healing	In 25 Wistar rats, 50 radial bone defects were created. In each group, there are 10 defects that were either left empty or filled at random with a combination of chitosan, gelatin and chitosan-gelatin autograft. After eight weeks, the rats were subjected to death and the animals were clinically and radiologically.	Digital radiographs: lateral surface of the injured healing radial bones. Bone volume/total volume: 3D CT. H&E staining: new bone formation ANOVA one-way Tukey post-hoc	In respect to bone regeneration, there were no discernible differences between the gelatin and gelatin-chitosan groups (P > 0.05). Gelatin, either applied alone or in conjunction with chitosan, was found to have positive effects in bone regeneration and can be a useful tool for bone tissue engineering applications.
Zang et al. (2016) [16]	In vitro and in vivo study (6 beagle dogs n=3)	Group 1: chitosan + bone marrow-derived mesenchymal stem cells seeded scaffold; Group 2: normal wound healing	Six beagles underwent surgery to create one-wall infrabony defects at the bilateral mandibular third premolars and first molars. Six groups were randomly assigned to implant the defects using different scaffolds: ABB scaffold (ABB), hJBMMSCs (C/ABB+cell), C/ABB scaffold (C/ABB), chitosan scaffold (C), chitosan scaffold with hJBMMSCs (C+cell) and open flap debridement (control). The animals were put down for histological examination eight weeks after the procedure.	Bone volume/total volume: micro-CT analysis H&E staining and Masson’s trichrome staining- new bone formation Immunohistochemistry- ELISA for osteocalcin protein is more in group 1 one-way ANOVA post-hoc Kruskal-Wallis test	Group C/ABB+cells formed cementum at a significantly higher rate than group C/ABB (2.64±0.50 mm versus 0.91±0.55 mm, P<0.05). Groups C/ABB+cell and C/ABB displayed mean±(standard deviation) values for new bone height and area of 2.89±1.65 and 8.73±1.94 mm^2^ respectively 2.83±0.29 and 2.65±0.52 mm. There was no discernible difference in new bone (area and height) between the two groups.
Zhou et al. (2017) [17]	In vitro and in vivo study (rat calvarial defects n=30)	Group 1: chitosan + Whitelock; Group 2: chitosan + hydroxyapatite; Group 3: vacant defects	We used thirty male rats. The porous scaffolds were divided into a disk that measured 5 mm in diameter and 1.5-2 mm in thickness. The bone defects were separated into three groups and either WH/chitosan porous scaffold (n=12), HAP/chitosan porous scaffold (n=12), or empty (n=6) (negative control) was employed to fill the defects at random.	1. Bone volume/total volume: micro-CT analysis 2. Fluorescent labelling and H&E staining: new bone formation t-test and one-way analysis variance were done.	The findings showed that compared to HAP/chitosan, the WH/chitosan composite membrane exhibited higher osteogenic differentiation proliferation of human mesenchymal stem cells. Furthermore, the porous WH/chitosan scaffold has the potential to greatly enhance bone regrowth.
Sukpaita et al. (2019) [18]	In vivo study (rat calvarial defects)	Group 1: empty defects; Group 2: defects with chitosan; Group 3: defects with chitosan and dicarboxylic acid	To assess the CS/DA scaffold’s ability to regenerate bone in mice with calvarial defects with and without seeded primary human periodontal ligament cells (hPDLCs). Three groups consisting of 18 mice each were given either empty defects, defects with CS/DA scaffold or defects with both hPDLCs and CS/DA scaffold.	1. Bone volume/total volume: micro-CT analysis 2. H&E staining: new bone formation; bone volume increased in group 3: micro-CT analysis H&E staining shows new bone formation in group 3. ANOVA and independent t-test.	After six and 12 weeks, micro-CT demonstrated that CS/DA scaffolds markedly enhanced in vivo bone regeneration. According to histological analysis, both with and without hPDLCs, new bone formation was detected in defects with CS/DA scaffold.

Results

A total of 195 items were initially found in the electronic research conducted, which were then carefully analysed. Various articles were excluded based on the relevance of the title and abstract of a topic, articles published before 2010 and articles which did not meet the inclusion criteria. A total of six studies were included in the study which were in vivo studies. The chitosan and gelatin in conjunction with other biomaterials and molecules or CRS1 cells such as hydroxyapatite (HA), gelatin (G), collagen, hyaluronic acid (Ha), dicarboxylic acid (DA) and Whitelock (WL) were used in the studies that were chosen. Alkaline phosphatase activity (ALP) and bone volume measurements are included in the study results (Tables 4-5).

**Table 4:** Micro-CT analysis in various studies CFB-HAP: chitosan/fibroin-hydroxyapatite; hPDLCs: human periodontal ligament cells; micro-CT: micro-computed tomography; HAP: hydroxyapatite; WH: Whitelock

Study type	Group	Bone regeneration measurement	Results
In vitro	i) CFB-HAP membrane	Micro-CT analysis at two, four, and eight weeks post-surgery	i) 164.18±18.09
	ii) Resorbable collagen (bioguide)		ii) 158.32±24.38
	iii) No membrane used		iii) 147.36±9.85
In vitro	I) Chitosan scaffold	Micro-CT analysis at eight weeks post-surgery	i) 60.3±0.01
	ii) Gelatin scaffold		ii) 53.8±48.208
	iii) Chitosan-gelatin scaffold		iii) 108±0.05
	iv) Empty defects		iv) 42.3±10.80
In vitro	I) HAP/chitosan scaffold	Micro-CT analysis at eight weeks post-surgery	i) 49.3±0.03
	ii) WH/chitosan scaffold		ii) 58.4±0.05
In vitro	I) Empty defects	Micro-CT analysis at six and 12 weeks	i) 29.3±12.01
	ii) Defects with chitosan/dicarboxylic acid scaffold		ii) 34.8±10.08
	iii) Defects with chitosan/dicarboxylic acid scaffold with hPDLCs		iii) 26.9±13.48

**Table 5:** ALP activity in various studies CFB-HAP: chitosan/fibroin-hydroxyapatite; HAP: hydroxyapatite; WH: Whitelock; ALP: alkaline phosphatase activity

Type of study	Parameters	Alkaline phosphatase activity (ALP)	Results
In vivo [13]	I) CFB/HAP membrane	ALP of osteoblasts on different defects after 45^th^ day (U/gprot = unit/gram protein)	i) ALP protein concentration = 46 U/gprot
	ii) Resorbable collagen		ii) ALP protein concentration = 42 U/gprot
	iii) No membrane used		iii) ALP protein concentration = 38 U/gprot
In vivo [16]	I) HAP/Chitosan	ALP of chitosan at 45^th^ day (U/gprot = unit/gram protein)	i) ALP protein concentration =32 U/gprot
	ii) WH/Chitosan		ii) ALP protein concentration = 50 U/gprot

This systematic review includes six studies in total, out of which one study is the in vivo study, and the other five are both in vitro and in vivo studies. All have studied chitosan-based scaffolds incorporated with various biomolecules to evaluate bone regeneration. The main parameter evaluated in all six studies was the bone volume or total volume using micro-CT analysis. According to these studies, the chitosan and gelatin-based scaffold was effective in bone regeneration.

Immunohistochemistry done by Ge et al. [13] using STRO-1 (MSC marker) showed the highest bone growth in periodontal ligament stem cells (PDLSCs) and a seeded nano hydroxyapatite-coated-genipin-chitosan conjunction scaffold (HGCCS). In the studies done by Song et al. [14] and Oryan et al. [15], the regeneration in the calvarial bone of mice was evaluated with the help of micro-CT analysis and ALP analysis, which showed the chitosan/fibroin-hydroxyapatite (CFB-HAP) membrane had the highest bone volume (164.18±18.09) when compared to others, whereas empty defects have shown the least bone volume (29.3±12.01) in CT analysis, whereas the ALP showed that the CFB-HAP membrane had the highest ALP activity (49 µ/g protein) when compared with others. Masson’s trichrome staining used by Zang et al. [16] showed an improved new bone formation in human jaw bone marrow-derived mesenchymal stem cells (hJBMMSCs) that incorporated chitosan/anorganic bovine bone (C/ABB) scaffold. Zhou et al. [17] have seen enhanced bone regrowth in whitelock chitosan (WH/chitosan) scaffold when compared with hydroxyapatite chitosan (HAP/chitosan) scaffold with the help of hematoxylin and eosin (H&E) staining. The study done by Sukpaita et al. [18] demonstrated marked bone regeneration with the primary human periodontal ligament stem cells (hPDLCs) incorporated chitosan/dicarboxylic acid (CS/DA) scaffolds in the micro-CT analysis. The average duration for evaluating bone formation with micro-CT was six to 12 weeks. The edges of the defect displayed mineralised tissue that resembled bone growth in the histologic sections of the undecalcified sections (stained with von Kossa).

Discussions

The systematic review evaluates the effectiveness of chitosan and gelatin-based scaffolds for bone regeneration in periodontal intrabony defects. Various parameters of measurement such as bone volume/total volume using micro-CT analysis, ALP and H&E-stained sections of mice calvaria have been evaluated for new bone formation. It also highlights the advantage of bilayered and trilayered chitosan and gelatin-based scaffolds combined with biomaterials in promoting the regeneration of different tissues in the periodontium.

Chitosan and gelatin are versatile biomaterials with numerous applications in the field of tissue engineering. These biomaterials are used in creating scaffolds that provide a three-dimensional structure that mimics the natural ECM of tissue, supporting the growth and regeneration of PDL cells [19]. Both chitosan and gelatin are biocompatible and biodegradable, and have the ability to promote cell adhesion and migration facilitating tissue growth and repair [20]. Research involving the incorporation of chitosan with different types of stem cells, such as hJBMMSCs, hPDLCs and PDLSCs revealed a noteworthy rise in the expression of genes related to osteoblasts, ALP and the upregulation of markers related to bone, including osteo sialoprotein, osteopontin and osteocalcin when observed in vitro [21]. Following six and 12 weeks of in vivo studies, micro-CT and histology revealed substantial calvarial repair and new bone formation. It can be said that while chitosan acts as a carrier for stem cells and can be used on its own to treat bone defects, the addition of stem cells to chitosan can greatly enhance bone regeneration.

In the in vivo studies, chitosan aided with other materials such as DA, WH, ABB and fibroin-hydroxyapatite. Before the animals were put to sleep, radiography and clinical assessment were performed on them. Experimentally, critical-size radial bone defects were created in the rats, and these defects were filled with these materials. After the rats were put down after eight weeks, samples of their healing bone were taken, and radiography, CT scanning, biomechanical testing, gross pathology, histopathology, histomorphometry and scanning electron microscopy were used to assess the samples [22].

Chitosan by itself was unable to promote new bone formation indicating superior osteoinductive and osteoconductive properties of the incorporated scaffolds [23]. The implantation of different materials with chitosan scaffolds increased new bone formation and mechanical properties compared with the untreated defects in rats [24]. Literature evidence suggests that the chitosan solution promoted the synthesis of type I collagen, which is a key component of bone tissue and facilitated the differentiation of hPDLs into osteoblasts [25]. The study done with the WH pellets combined with chitosan has the ability to enhance the proliferation and osteogenic differentiation of osteoblasts creating a favourable microenvironment for osteoblast activity [26]. The in vivo study using a rabbit model demonstrated that the controlled release of GFs like BMP-2 and insulin-like growth factor-1 (IGF-1) from chitosan scaffold was a therapeutic approach to promote bone healing and regeneration [27].

The findings from the included studies and previous literature show that, when used individually for bone regeneration, the biomaterials, chitosan and gelatin have various disadvantages such as limited strength, rapid degradation, immunogenicity and limited versatility. This systematic review revealed that the combination of these two materials showed enhancement in properties such as improved tensile strength, slower degradation rate and controlled drug delivery along with improvement in the biocompatibility of the scaffold. This enhancement contributed to significantly greater new bone formation in the chitosan-gelatin group compared to the chitosan-alone group.

Limitations

Even though all the studies included in the review showed bone regeneration with chitosan and gelatin-based scaffolds, only in vivo and in vitro studies were included in the systemic review and are considered as a low level of evidence.

Clinical Significance

Based on the results of this systematic review, it is seen that the chitosan and gelatin-based scaffold was effective in bone regeneration, and this property can be considered in the development of chitosan and gelatin-based scaffolds, which can be used further in human clinical trials, especially in the treatment of periodontal intrabony defects where selective repopulation of the cells is required.

## Conclusions

The systematic review concludes that the bone regeneration using chitosan and gelatin-based scaffolds showed significant bone volume; total volume was seen at the end of 12 weeks and the histology sections showed collagen formation and mineralisation of the bone. This scaffold has shown preliminary evidence that it carries the potential to induce bone regeneration, and these scaffolds can be further considered for clinical evaluation.
